# A cross-correlator-based timing tool for FemtoMAX

**DOI:** 10.1107/S1600577525003479

**Published:** 2025-06-02

**Authors:** D. Kroon, E. Nilsson, B. Ahn, M. Bertelli, R. Calarco, I. Clementsson, S. De Simone, J. C. Ekström, A. Jurgilaitis, M. Longo, V. T. Pham, J. Larsson

**Affiliations:** a MAX IV Laboratory Lund University PO Box 118 SE-221 00Lund Sweden; b Department of Physics Lund University PO Box 118 SE-221 00Lund Sweden; c Institute for Microelectronics and Microsystems (IMM) Consiglio Nazionale delle Ricerche (CNR) Via del Fosso del Cavaliere 100 00133Rome Italy; d Department of Chemistry University of Rome Tor Vergata Via della Ricerca Scientifica 1 00133Rome Italy; e LINXS Institute of Advanced Neutron and X-ray Science, Mesongatan 4, 224 84Lund, Sweden; ESRF – The European Synchrotron, France

**Keywords:** femtosecond laser, synchronization, time-resolved pump/probe experiments, timing tool, timing jitter monitor

## Abstract

We report on the commissioning of an ultrafast timing diagnostic for measuring a time-offset signal between two different synchronized ultrashort light pulses. The method is based on sum-frequency generation in a nonlinear crystal. The setup is similar to an auto/cross-correlator setup. In this case, one of the beams is a relatively weak diagnostic beam of visible light from a bending magnet at FemtoMAX (10^6^ photons pulse^−1^ in a 0.2 mm high and 5 mm wide beam) while the other is a relatively intense laser beam (200 µJ pulse^−1^) derived from the same laser that is used to pump the sample in pump/probe experiments. This enables online monitoring of the relative timing of a linear accelerator-based, short-pulse, hard X-ray source and a synchronized visible laser. We show that for a <50 fs full width at half-maximum (FWHM) light pulse from the accelerator and a 50 fs (FWHM) long laser pulse, we can determine the relative timing of the two pulses with an accuracy below 30 fs in a time interval of 4 ps. The advantages and limitations of the method are discussed.

## Abstract 2

A novel timing tool suited for FemtoMAX is described. This will allow sub-100 fs time-resolved measurements at FemtoMAX.

## 1. Introduction

Laser-based, time-resolved pump/probe experiments using accelerator-based sources for probing are important tools for investigating structural dynamics in materials. The accuracy for synchronization has gradually improved, but, for both feedback and for post-sorting of data, it is important to measure the time shift between pump and probe pulses with high temporal resolution. In the context of measuring the position of atomic nuclei as a function of time, this means a temporal resolution significantly below 100 fs. Studies of structural dynamics on this timescale are commonly performed using time-resolved optical pump/hard X-ray probe methods (Lindenberg *et al.*, 2017 ▸; Buzzi *et al.*, 2018 ▸; Trigo *et al.*, 2013 ▸; Daranciang *et al.*, 2012 ▸). The first femto­second X-ray sources used laser-driven plasma sources (Sokolowski-Tinten *et al.*, 2003 ▸) or slicing in storage rings (Zholents & Zolotorev, 1996 ▸; Schoenlein *et al.*, 2000 ▸). Both these types of sources are inherently synchronized, provided that the drifts/jitter in the transport of the laser beams can be avoided. The high temporal resolution using relatively low flux was demonstrated in several experiments (Johnson *et al.*, 2003 ▸; Johnson *et al.*, 2008 ▸). Experiments using higher X-ray flux per pulse are carried out at hard free-electron laser (FEL) facilities, such as Linac Coherent Light Source (LCLS) (Moeller *et al.*, 2011 ▸), SACLA (Yabashi *et al.*, 2015 ▸), SwissFEL (Milne *et al.*, 2017 ▸), European XFEL (Altarelli, 2015 ▸) and other LINAC-based, short-pulse X-ray facilities such as FemtoMAX at MAX IV Laboratory (Enquist *et al.*, 2018 ▸).

It is challenging to obtain a timing jitter which is lower than about 200 fs (FWHM) for a LINAC-based source, but the timing tools enable accuracy in the 10 fs range. The first timing monitors to be implemented were based on electro-optic sampling (EOS) (Cavalieri *et al.*, 2005 ▸). They were developed and implemented at the decommissioned Sub-Picosecond Pulse Source (SPPS) at SLAC and at FLASH at DESY (Hoffmann *et al.*, 2011 ▸). EOS relies on a replica of the pump laser pulse being sent from the laser into the proximity of the electron beam dump. The field generated from the relativistic electrons can induce birefringence in a crystal which rotates the polarization of the replica pump pulse if they are overlapped in time. Spectral or spatial chirps can be employed in order to obtain a single-shot measurement of the jitter. The path length of a beam going into the accelerator complex to overlap in time with the electron pulse that generated the X-ray pulse can be quite long. That means that the path length difference compared with a beam going directly from the laser to the sample is substantial. At SPPS the beam was sent nearly 100 m into the accelerator tunnel, whereas the laser beam was transported less than 10 m to the experiment. For that reason, a previous oscillator pulse is often used for timing monitoring rather than building 100 m-long delay lines. That still makes the setup sensitive to drifts of path lengths in the laser amplifiers and the two long beam paths.

At FELs, which have a high flux, it is possible to split off a small fraction of the FEL beam, which can be used as a diagnostic beam. This enables accurate and now standardized methods where this diagnostic beam pumps a material. This leads to the generation of free carriers which influence the reflection and transmission of a visible replica pump pulse beam. A chirped visible pulse can be used together with a spectrometer in order to encode the spectrum to time and accurately derive the temporal jitter in a single shot. 1 × 10^10^ photons per pulse in a 0.5 mm spot gives a 10% change in the spectral encoding signal (D. Zhu, private communication).

At comparatively low-flux sources, such as FemtoMAX (Enquist *et al.*, 2018 ▸), there are not enough X-ray photons to achieve a measurable visible reflectivity change. Instead, we have used the single-shot cross-correlator optical scheme proposed by Enquist *et al.* (2018 ▸). The experiments were carried out at the FemtoMAX beamline at MAX IV and the setup is intended as a timing tool for regular user operation.

We mix a small fraction of the laser pulse with visible bending magnet radiation from the dump magnet. The angular acceptance of the extraction system is small (2 mrad), which means that temporal broadening effects are below 1 fs, and the bending magnet radiation has the same shape and duration as the electron bunch. The X-ray pulse shape will be identical to the electron bunch shape for an incoherent source, such as FemtoMAX.

The cross-correlator device is similar to a single-shot auto-correlator. The possibility to measure the pulse width of ultrashort optical radiation utilizing nonlinear optical techniques was first proposed by Weber (1967 ▸). A method to characterize short light pulses using nonlinear correlation techniques was proposed theoretically by Janszky *et al.* (1977 ▸). The ability to synchronize two laser sources by using nonlinear optics is described and implemented by, for example, Ma *et al.* (2001 ▸). A precise timing signal can either be used to individually timestamp each probe pulse relative to the pump pulse or be used as feedback in a phase-locked loop circuit which would enable accurate control of the relative timing of two different sources. At an X-ray facility such as FemtoMAX, the laser is locked to the phase of the radio-frequency (RF) wave which accelerates the electrons. However, the electron pulse generally has non-negligible jitter/drift relative to the RF (Craievich *et al.*, 2013 ▸). At FemtoMAX we observe jitter between X-ray and laser of up to 1 ps and drifts of up to 5 ps over several hours.

## 2. Experimental setup

The FemtoMAX beamline (Enquist *et al.*, 2018 ▸; Wang *et al.*, 2020 ▸) has been designed for laser pump/X-ray probe measurement on the 100 fs timescale. Since the first description of FemtoMAX (Enquist *et al.*, 2018 ▸), several improvements have been made. The repetition rate has now been increased to 10 Hz. Two 5 m in-vacuum undulators were installed and commissioned in 2018. The electron beam has been improved to a diameter of 150 µm, which is demagnified to an X-ray beam size of 60 µm (FWHM). This beam size can be achieved at the sample position for all commissioned endstations. Furthermore, a transverse deflecting cavity has been installed in the accelerator complex and a pulse duration of 50 fs (FWHM) for the electron bunch has been measured.

The laser system at FemtoMAX is a Ti:sapphire amplifier (KM Laboratories Red Wyvern). It can run at a repetition rate up to 100 Hz and delivers pulses with a duration of 50 fs (FWHM) and pulse energy of 11 mJ at a center wavelength of 795 nm. The laser system also includes an optical parametric amplifier (TOPAS HE from Light Conversion) with mixing stages to cover the 0.2–10 µm wavelength range. The laser system is situated one floor above the experimental hutch. In the laser laboratory the laser beam is split into two separate arms: the diagnostics arm, carrying about 10% of the fundamental laser power, and the pump arm. Both the pump arm and the diagnostics arm are about 15 m long and contained in two separate vacuum transport systems. The pump arm has six motor-controlled interchangeable mirrors (Al, Au and dielectric 760–840 nm mirrors) for sample pumping in different spectral regions, whereas the diagnostic arm uses dielectric 760–840 nm mirrors. The cross-correlation timing monitor was implemented in the diagnostics arm, which is also used for two other timing monitors with lower temporal resolution, an RF cavity-based timing monitor and a streak camera. In this paper, we describe the cross-correlation timing monitor.

**Figure 1:**
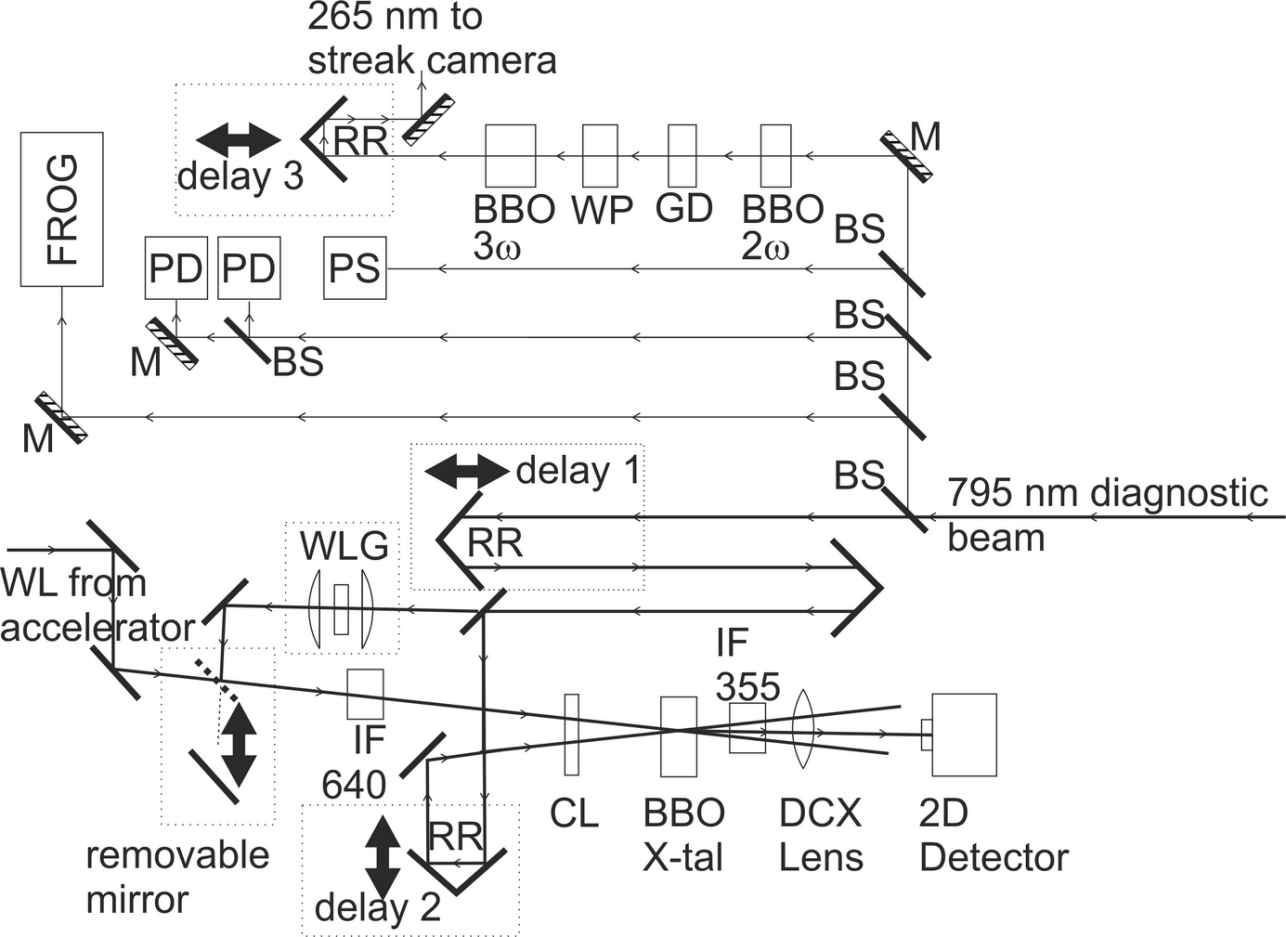
Overview of the timing diagnostics. The lower part with bold lines shows the cross-correlator, including a path to create laser-generated white light in order to set up the sum-frequency generation process. The interference filter is generally only used for setup. The FROG is for monitoring the temporal amplitude and phase of the laser pulse. The photodiodes (PD) are used for the RF-based timing monitor. A UV pulse is generated for an X-ray streak camera that is triggered by a photo-conductive switch. The abbreviations in the figure are as follows. RR: retroreflector; WLG: white-light generation; WL: white light; BBO: beta barium borate non-linear crystal; 2ω indicates frequency doubling and 3ω indicates mixing of the fundamental and the doubled radiation; DCX: double convex lens; CL: cylindrical lens; 2D: two-dimensional detector; BS: beam splitter; M: mirror; FROG: frequency-resolved optical gating; WP: wave-plate; GD: group delay compensation plate; PD: photodiode; PS: photo-conductive switch; IF355: 355 nm interference filter used to select the cross-correlator signal.

The diagnostic setup is placed about 4 m upstreams of the endstation, and the edge of the setup is only offset 20 cm from the X-ray beam path. When the diagnostic beam reaches the diagnostic setup it is split into several beams. One is used for measuring the beam characteristics in a frequency-resolved optical gating (FROG) setup. A second beam is used for an RF-based timing monitor which is described briefly below and will be described in detail elsewhere (Kroon *et al.*, 2025 ▸). A third beam is used for a combined X-ray/UV-laser streak camera that is under commissioning and two beams which are used to trigger two separate photo-conductive switches for the streak camera. There are two more beams used for the cross-correlator. One of the beams is the mixing beam used together with the light from the bending magnet, and the last beam can be used to generate white light in a fused silica glass plate that can be used for setup and calibration of the cross-correlator when the light from the bending magnet is not available. A sketch of the diagnostic table is shown in Fig. 1 ▸.

**Figure 2:**
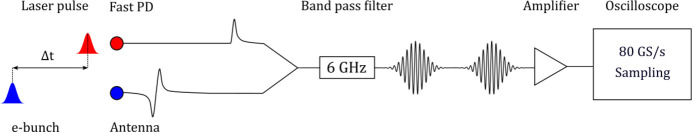
Schematic of the RF-cavity based timing tool at FemtoMAX which was used as a reference in this study. The electron bunch interacts with a button antenna, which also acts as a beam position monitor. The laser is detected by a fast photodiode (Fast PD). The two signals are combined and excite an RF cavity resonance. The two signals are amplified and measured on the same oscilloscope channel using a 80 Gs s^−1^ oscilloscope.

The timing monitor based on RF cavities utilizes two fast signals derived from the laser pulse and the electron pulse, respectively, as illustrated in Fig. 2 ▸. The electron pulse signal is picked up from one of two button antennas located next to the vacuum tube downstream from the FemtoMAX undulators. A broadband RF splitter/combiner is used to combine the signal from the antenna with a signal generated by a laser pulse detected by a fast photodiode (PD in Fig. 1 ▸) (12 GHz analog bandwidth, 35 ps FWHM). The combined signals then excite a 6 GHz RF cavity band-pass filter that rings for about 10 ns (Fig. 2 ▸). The ringing signals are amplified and sampled at 80 Gs s^−1^ by a 36 GHz bandwidth oscilloscope.

A Hilbert-transform based algorithm is used to extract the relative time between the two signals exciting the RF cavity. Two replicas of this setup are run in parallel and averaged in order to reduce the statistical measurement error. For that reason, Fig. 1 ▸ has two photodiodes (PD). The accuracy of the timing measurement has been demonstrated to be 200 fs (FWHM) in an experiment where the scattering intensity of the Bi (111) reflection was observed following excitation of the A_1_ phonon mode (Sokolowski-Tinten *et al.*, 2003 ▸; Fritz *et al.*, 2007 ▸). The time range of this timing monitor tool exceeds 100 ns. Since it builds on exciting (pinging) a band-pass filter it is referred to as the ‘ping timing monitor’ or simply the ‘ping’.

**Figure 3:**
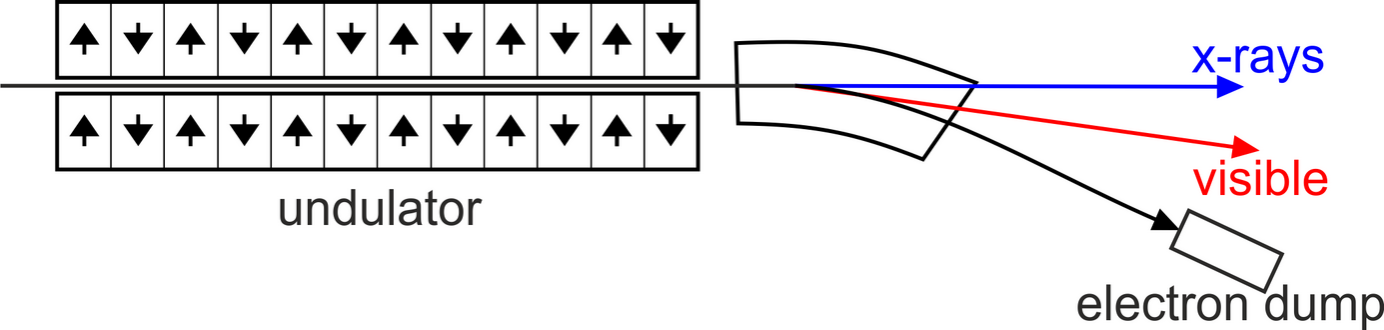
Conceptual set-up for extraction of visible light from the dump magnet of the LINAC.

Laser pulses with a duration of 50 fs and energy of approximately 1 mJ are available as a diagnostics beam. The central wavelength of the Ti:sapphire laser system is 795 nm and the spectral FWHM is 25 nm. The beam diameter is 10 mm. A schematic view of the diagnostics table can be found in Fig. 1 ▸. The extraction of visible light is illustrated in Fig. 3 ▸.

The two beams are vertically focused with cylindrical lenses (CL in Fig. 1 ▸) in order to increase the intensity of the signal on the camera. The beams are spatially overlapped in the interaction region of a pre-aligned nonlinear crystal [the beta barium borate (BBO) crystal was cut for non-collinear type 1 sum frequency generation of a 640 nm and a 795 nm beam at a relative angle of 11° outside the crystal which is 7° inside the crystal due to refraction at the surface]. The energy of the laser pulse at 795 nm was 200 µJ. The size of this laser beam is 10 mm × 0.5 mm inside the crystal. The weak beam from the bending magnet has a pulse energy down to ∼3 × 10^−13^ J, corresponding to ∼10^6^ photons pulse^−1^ mm^−2^. The size in the BBO crystal is 0.2 mm × 5 mm. The interaction region in the crystal is imaged onto the detector in order to avoid diffraction broadening. The 3:2 imaging optics consists of a 25 mm-diameter, UV-grade fused silica, 50 mm focal length lens placed at a distance of 130 mm from the crystal. The sum-frequency generation (SFG) light passes through a metallic tube placed between the lens and the detector in order to minimize the surrounding light background. A 355 nm interference filter is placed before the imaging lens (DCX in Fig. 1 ▸).

**Figure 4:**
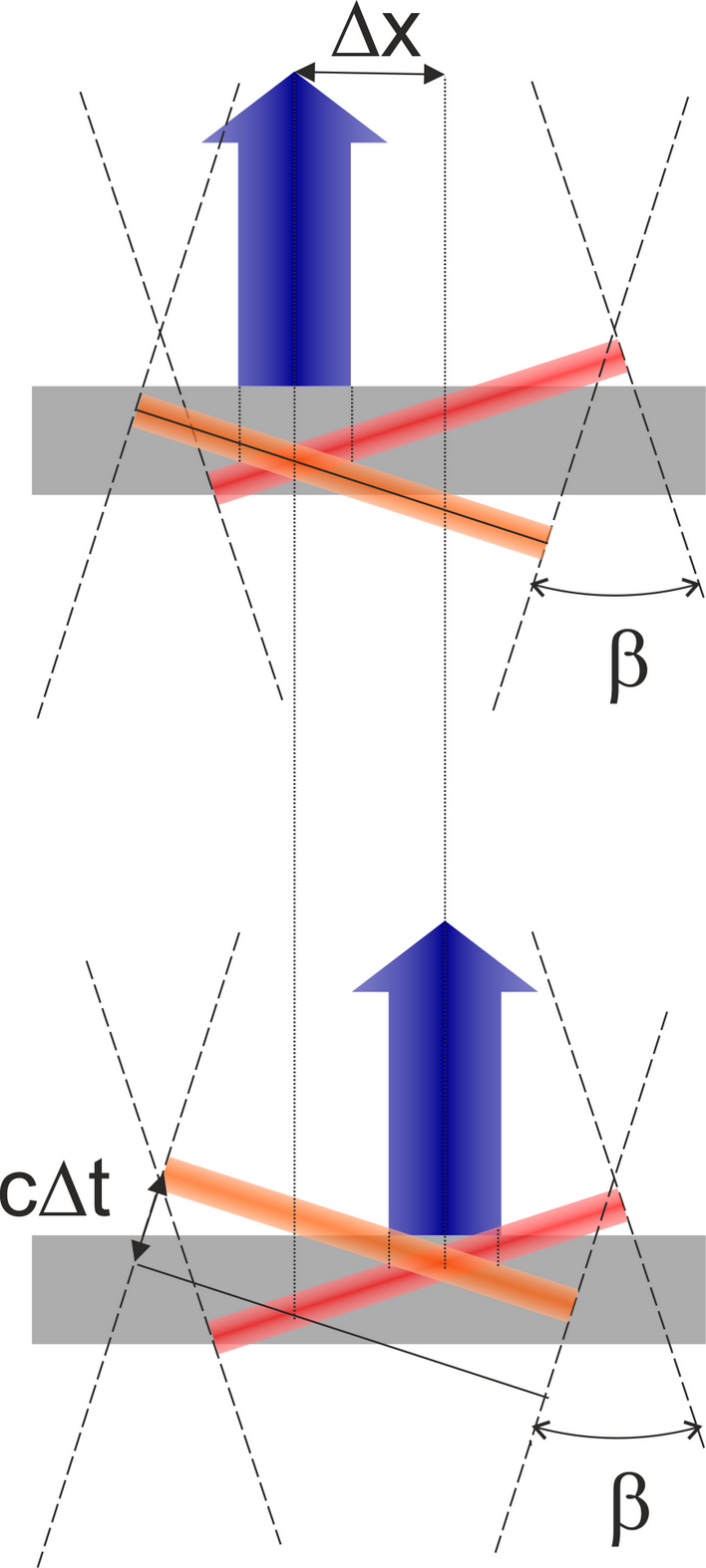
The SFG signal generated for two different time delays between the weak pulse from the bending magnet (orange) and the intense femtosecond light-pulse from the laser (red) is marked as a blue arrow. In the lower image the bending magnet pulse arrives sooner by a distance of *c*Δ*t* resulting in a change of position of the generated signal (blue arrow). After calibration, the position of the SFG signal is a direct measure of the relative time between the laser pulse and the light pulse from the bending magnet.

In the interaction region, the spatial width of the sum-frequency signal corresponds to the duration of the convolution of the pulses; the spatial position corresponds to the delay between the pulses as illustrated in Fig. 4 ▸. The signals measured are shown as blue arrows with the origin from the horizontal position within the crystal (gray) where the peaks of the two pulse fronts intersect. These measurements were made using an image intensifier relay imaged (3:2) onto a scientific CMOS camera (Zyla 5.5M from Andor).

There is a constant background from the detector, which is acquired at the beginning of the experiment and subtracted. Otherwise, the background is low except for random events when γ-rays or cosmic rays hit the sensor. Over a range of 4 ps, the SFG intensity does not depend on the relative timing of the two light pulses, but the interaction takes place in different lateral positions as shown in Fig. 4 ▸.

The time shift resolution in this method is limited by the pulse duration and the signal-to-noise (S/N) ratio. If the S/N ratio is high, the time shift resolution can be a small fraction of the cross-correlation width of the pulses. Geometrically the FWHM of the SFG generating region can roughly be expressed as
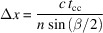
where *c* is the speed of light, *t*_cc_ denotes the cross-correlation width, *n* is the refractive index of the nonlinear crystal and β is the angle between the two beams.

**Figure 5:**
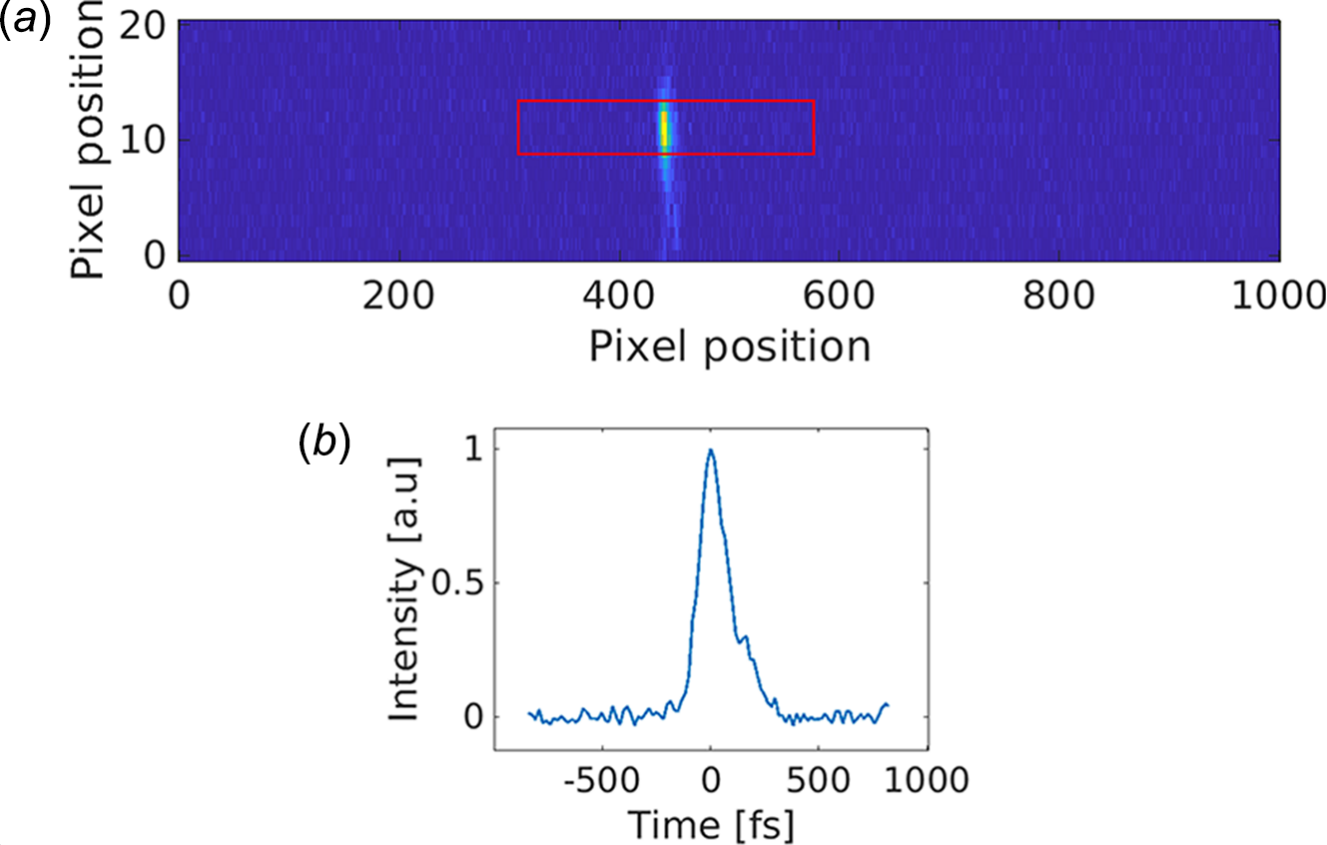
(*a*) Raw data from the 2D CCD detector where the strong feature is the cross-correlator signal. The background has been subtracted. A height of about 10 pixels correspond to 0.2 mm and the width of the beam is a measure of the temporal convolution of the pulse-shapes of the laser and the bending magnet radiation. The horizontal position of the signal is a measure of the relative timing of the electron beam and the laser. The calibration factor is approximately 16 fs per pixel. (*b*) Horizontal lineout of the cross-correlator signal in (*a*) calibrated in femtoseconds.

The raw data in Fig. 5 ▸(*a*) shows the full delay interval marked in red, which equals Δ*t* = 4 ps. The accuracy of the time-offset information depends on the spatial resolution of the detector, whereas the full time-offset interval is proportional to the full dimension of an intersection region of two beams on the nonlinear crystal.

The facility operates at 10 Hz, and all components at the beamline including the LINAC are designed for 100 Hz operation. The method itself is much faster and could be employed at a different facility at a repetition rate of at least 1 kHz. This limit is due to the CCD detector.

## 3. Alignment

In order to align the cross-correlator, the removable mirror in Fig. 1 ▸ is set to the in-position which enables the use of the perfectly timed, and very intense, laser-induced white light. The pathlengths between the 795 nm beam and the laser-induced white light are aligned to be equally long (within 10 ps = 3 mm) using a fast photodiode. The path length of the 795 nm beam is set by mixing any color that fulfils the phase-matching condition. When the full intensity of the laser-generated white light is used, the mixing signal around 358 nm in the form of a vertical line can be seen by the naked eye on a card. Subsequently, the phase-matching angle for 640 nm is found by optimizing the angle of the BBO crystal while viewing the cross-correlator signal behind a 355 nm interference filter. Following this step, the white-light intensity is reduced in steps by up to six orders of magnitude in order to reach the same level as the white light derived from the electron pulse. The detection system consisting of imaging lenses, interference filters, image intensifier and camera is set up and the signal is optimized. A second camera looks at the scattered light from the crystal (both white light and laser). The white light from the accelerator has a wide spectrum and can also be seen and overlapped on the crystal. Following this step, only the timing needs to be scanned electronically in order to find the signal. To find the right scan range a fast diode or microchannel-plate photomultiplier tube (MCP-PMT) can be temporarily positioned near the plane of the BBO crystal. It allows us to find the correct timing to within 30 ps.

## 4. Measurements

The incident number of photons in the white light pulse within the 20 nm-wide phase-matching range is measured by inserting an interference filter at 650 nm and measuring the signal on the MCP-PMT positioned behind the filter (IF 640 in Fig. 1 ▸). The quantum efficiency of the MCP photocathode, the charge of an electron and the gain of the MCP are known, which makes it straightforward to derive the number of photons behind an aperture. We use 2 × 10^5^ photons at 650 nm, which is up-converted and filtered using a UV interference filter (IF 355 in Fig. 1 ▸) to 5 × 10^3^ photons at 358 nm. As the photocathode has a quantum efficiency of 20%, the average number of photons detected is 1 × 10^3^.

Scans were recorded where we measured the signal from the RF-based timing monitor at the same time as the cross-correlator signal. We also performed a laser-pump/X-ray probe experiment at the same time. The sample was a Ge_2_Sb_2_Te_5_ thin powder film where we measured the diffuse scattering near the 200 reflection.

## 5. Analysis

A dark background for the cross-correlator detector was acquired by capturing 75000 images without the cross-correlator signal and averaging these images. For each image captured with the cross-correlator signal this background is removed. The images are then cropped in the *y*-direction to only consist of the region of interest that contains the actual signal. Each image is then summed along the *y*-direction, and the pixel position for the pixel with the highest signal count is saved. A region of interest around this pixel is then cropped out, and the center of mass in this region is calculated. The *x*-position of this center of gravity is saved as the cross-correlator pixel position. To analyze the measurements from the cross-correlation signal we use the ping signal as a calibration tool. We use two channels in parallel for the ping timing tool to obtain a more accurate reading. The shot is discarded if the two channels differ too much. Sometimes one of the channels might give an erroneous reading, and we only use measurements where the recorded values from the two channels are within 2 ps of each other. Only 0.35% of all shots are discarded based on this criterion.

When performing this analysis, a few incorrect measurements are also made due to double peaks in the cross-correlator signal originating from cosmic rays or gamma-rays from the accelerator. These secondary peaks are significantly narrower than the actual cross-correlator signal. By fitting a Gaussian to the peaks and only selecting the peaks that are wider than 3.5 pixels we manage to sort out all of the anomalous measurements. We then sorted out 97 out of 13172 measurements which corresponds to 0.74% of the data.

To calibrate the pixel position on the camera to the actual time-delay between the two light pulses, we fit a second-degree polynomial to the pixel position as a function of the ping reading while varying the delay time over the full range of the cross-correlator. The second-degree term corresponds to a small correction, less than 1.5 pixels, in any part of the range compared with a purely linear fit. The calibration is between 16 and 17 fs pixel^−1^ over the 4 ps range.

## 6. Results

The width of the SFG signal is a measure of the duration of the convolution of the pulse from the accelerator with the laser pulse. In Fig. 5 ▸(*b*) the lineout of the raw data in Fig. 5 ▸(*a*) can be seen. The horizontal scale has been converted from pixels to femtoseconds. We measured the peak width to be 140 fs. The laser pulse is around 50 fs and the temporal width of the cross-correlator signal must be due to the white light from the bending magnet. As the white light travels from the source to the interaction point, it passes through vacuum windows and lenses. We estimate the chirp coefficient to be *b* = 2.8 × 10^−27^ s^2^ (Saleh & Teich, 2007 ▸), since we accept 20 nm from the phase-matching that would give a temporal width of about 140 fs after passing the chirp filter. When convoluting this with a 50 fs pulse we obtain 150 fs, which is close to the measured width of the peak which is 140 fs.

**Figure 6:**
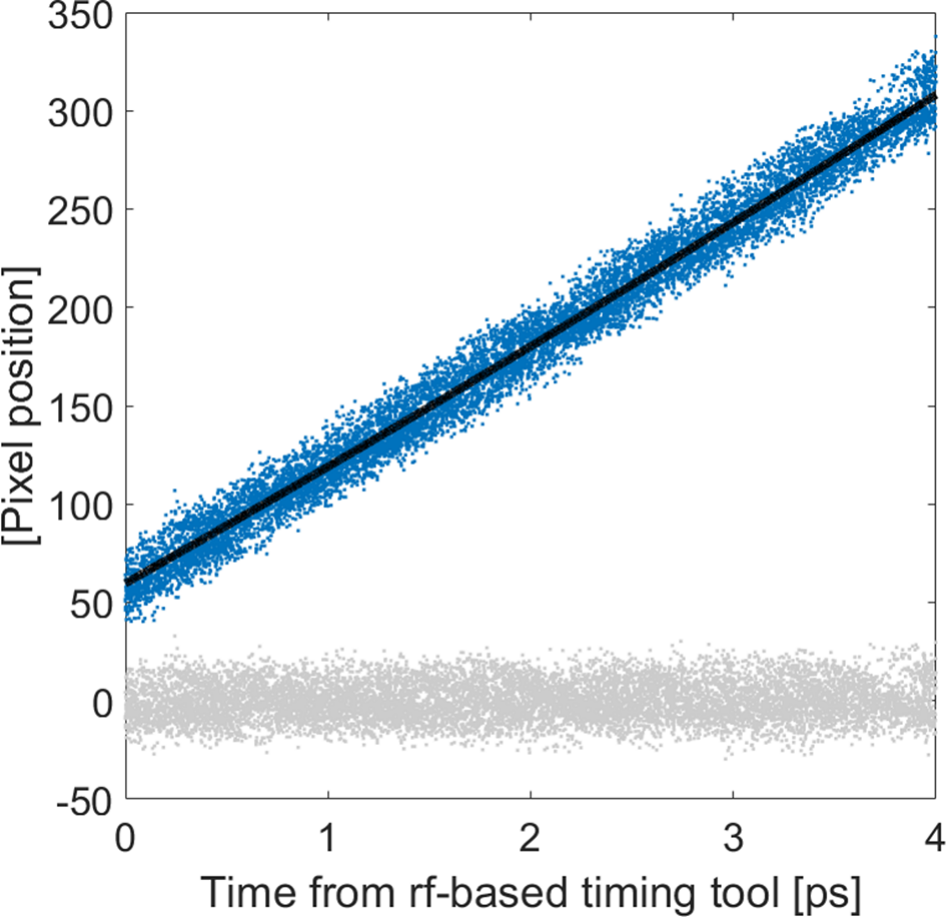
Data comparing the timing information from the ping timing monitor and the cross-correlator. These data are used for the temporal calibration. The noise is due to the 200 fs uncertainty of the ping. Ideally the fit would be linear, but a small second-order term has been added in order to obtain a flat residual over the full 4 ps. The residual, which is defined as the fit subtracted from the data, is plotted in gray.

As seen in Fig. 6 ▸, the temporal scale is nearly linear when compared with the well tested ping timing monitor.

**Figure 7:**
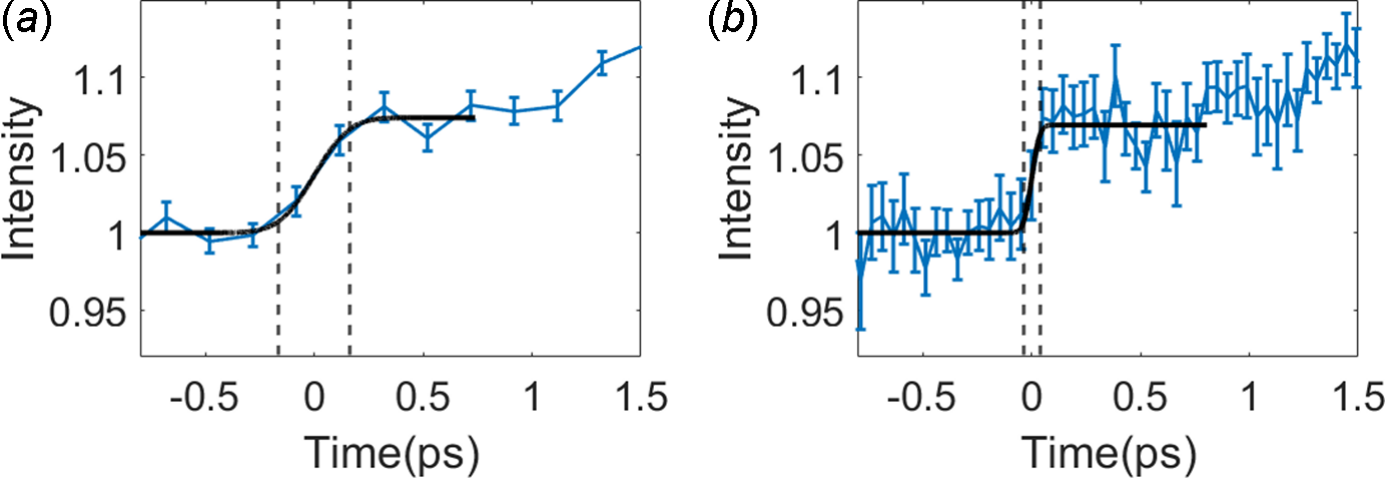
In (*a*) we see a rapid increase of the diffuse scattering from laser-excited Ge_2_Sb_2_Te_5_. The data are sorted by an RF-based timing tool. In (*b*) the speed of the dynamics is revealed to be significantly faster when the data have been sorted by the ultrafast cross-correlator timing tool. In both cases an error function, shown in black, has been fitted to the rapid rise. Using the RF-based ping monitor the 10% to 90% rise time of the error function is 330 fs while the corresponding time for the cross-correlator data is 70 fs.

The cross-correlator was commissioned in an experiment where the initial motion of atoms in the crystalline phase of the phase-change memory material Ge_2_Sb_2_Te_5_ was investigated. The initial dynamics of the diffuse scattering in a wide range around *Q* = 2.5 Å^−1^, near the 200 peak, is shown with the shots sorted by the ping timing tool and sorted by the cross-correlator. We attribute the rapid increase of the diffuse scattering to bond breaking and subsequent atomic motion. The result is remarkable. As can be seen in Fig. 7 ▸(*b*), the dynamics can be seen to occur within 70 fs, whereas the corresponding increase of intensity appears to have a time constant of about 330 fs using the ping timing tool. This opens up for the future study of ultrafast dynamics at FemtoMAX.

## 7. Discussion and conclusions

We have developed and tested a technique that is capable of providing temporal jitter information between ultrashort light pulses. The possibility of obtaining the relative time delay between light pulses by non-collinearly phase-matched frequency mixing in nonlinear crystals has been demonstrated. The jitter information is extracted from the horizontal position of the optical frequency mixing signal on a 2D detector.

The time resolution in the experiment using the CCD detector was limited by the dispersion of the pulse from the bending magnet. The FWHM of the signal shown in Fig. 5 ▸(*b*) is 140 fs FWHM. However, the pixel position of the peak can be found with higher accuracy. In order to evaluate the data quality and our ability to determine the center position of the signal, we derived the position of a Gaussian fit and compared it with a center of gravity calculation. A noisy signal would give different arrival times for different analysis methods. We find that when evaluating the full data set the RMS deviation is 15 fs, which yields a 2σ value of 30 fs. This is consistent with an intuitive error estimate by looking at the data quality of Fig. 5 ▸(*b*). The accuracy is also consistent with the fact that we see the temporal resolution in the X-ray experiment increase from 330 fs to 70 fs (rise-time 10%–90%) when the cross-correlator is used for sorting. The convolution of the X-ray and laser pulses alone accounts for the width of 70 fs which means that the error of the cross-correlator is less than 30 fs. A future improvement of the time shift accuracy of the cross-correlator could be achieved by compensating for the group velocity dispersion (GVD). At present, we find the accuracy sufficient and have not compressed the pulse derived from the electron bunch.

We have shown that our technique allows photon fluxes down to ∼10^6^ photons mm^−2^, providing a femtosecond accuracy time-offset measurement for each pulse. We expect the timing measurement to be integrated into the control system so that it is available for general users. It should be noted that this method is a jitter monitor. In order to find the time when the laser and X-rays arrive simultaneously, other methods on standardized samples, *e.g.* Bi (Fritz *et al.*, 2007 ▸; Epp *et al.*, 2017 ▸) and InSb (Bengtsson *et al.*, 2020 ▸), can be used.
